# Support Needs as Perceived by Grandparent-Caregivers: A Qualitative Systematic Review

**DOI:** 10.1093/geroni/igab046.2942

**Published:** 2021-12-17

**Authors:** Schola Matovu, Deborah Whitley, Heather Young

**Affiliations:** 1 University of Utah College of Nursing, Salt Lake City, Utah, United States; 2 Georgia State University, Atlanta, Georgia, United States; 3 University of California Davis, Sacramento, California, United States

## Abstract

Caregiving can have adverse mental and physical health outcomes. Older grandparents who are primary caregivers for their grandchildren report multiple health conditions such as depression, anxiety, hypertension, cardiac disease and chronic fatigue, which are caused by or otherwise exacerbated by the caregiving demands. We conducted this qualitative systematic review to identify support needs that contribute to such poor health outcomes and as perceived by grandparent-caregivers for minor grandchildren. We searched relevant databases (PubMed, PyschINFO, CINAHL, and Social Work Abstracts) using terms such as: child rearing, parenting, child custody, grandparents, support needs, and caregiving. Studies were included for review if they were written in the English language; used only qualitative methods; and were published from January 1990 to January 2020. Included studies were critically appraised using the Critical Appraisal Skills Programme checklist. Data were extracted from these studies and synthesized using meta-ethnography. Of the 2828 studies identified, 58 studies from 12 countries met all inclusion criteria for review. Three main themes emerged from the review: 1) grandparent-caregivers’ personal needs, and 2) grandchildren needs. Both themes were further divided into subthemes of health (mental & physical), financial, social (interpersonal, cultural and environmental factors and services). Findings from this review have potential to: 1) inform design of comprehensive interventions and screening needed to address perceived support needs; and 2) identify gaps in and barriers to available support resources for older grandparent-caregivers. Further research is needed on comprehensive assessment of support needs and risk for poor health outcomes among grandparent-caregivers.

